# B-cell reconstitution after lentiviral vector–mediated gene therapy in patients with Wiskott-Aldrich syndrome

**DOI:** 10.1016/j.jaci.2015.01.035

**Published:** 2015-09

**Authors:** Maria Carmina Castiello, Samantha Scaramuzza, Francesca Pala, Francesca Ferrua, Paolo Uva, Immacolata Brigida, Lucia Sereni, Mirjam van der Burg, Giorgio Ottaviano, Michael H. Albert, Maria Grazia Roncarolo, Luigi Naldini, Alessandro Aiuti, Anna Villa, Marita Bosticardo

**Affiliations:** a San Raffaele Telethon Institute for Gene Therapy (TIGET), IRCCS San Raffaele Scientific Institute, Milan, Italy; b Pediatric Immunohematology and Bone Marrow Transplantation Unit, IRCCS San Raffaele Scientific Institute, Milan, Italy; c CRS4, Science and Technology Park Polaris, Pula, Cagliari, Italy; d Department of Immunology, Erasmus MC, University Medical Center Rotterdam, Rotterdam, The Netherlands; e Dr von Hauner Children's Hospital, Ludwig-Maximilians-University Munich, Munich, Germany; f Vita-Salute San Raffaele University, Milan, Italy; g Department of System Medicine, Tor Vergata University, Rome, Italy; h IRGB CNR, Milan Unit, Milan, Italy

**Keywords:** Wiskott-Aldrich syndrome, gene therapy, B cell, primary immunodeficiency, lentiviral vector, BAFF, B cell–activating factor, BM, Bone marrow, GT, Gene therapy, HD, Healthy donor, HSC, Hematopoietic stem cell, IVIg, Intravenous immunoglobulin, PB, Peripheral blood, SDF-1α, Stromal cell–derived factor 1α, VCN, Vector copy number, WAS, Wiskott-Aldrich syndrome, WASp, Wiskott-Aldrich syndrome protein

## Abstract

**Background:**

Wiskott-Aldrich syndrome (WAS) is a severe X-linked immunodeficiency characterized by microthrombocytopenia, eczema, recurrent infections, and susceptibility to autoimmunity and lymphomas. Hematopoietic stem cell transplantation is the treatment of choice; however, administration of *WAS* gene–corrected autologous hematopoietic stem cells has been demonstrated as a feasible alternative therapeutic approach.

**Objective:**

Because B-cell homeostasis is perturbed in patients with WAS and restoration of immune competence is one of the main therapeutic goals, we have evaluated reconstitution of the B-cell compartment in 4 patients who received autologous hematopoietic stem cells transduced with lentiviral vector after a reduced-intensity conditioning regimen combined with anti-CD20 administration.

**Methods:**

We evaluated B-cell counts, B-cell subset distribution, B cell–activating factor and immunoglobulin levels, and autoantibody production before and after gene therapy (GT). *WAS* gene transfer in B cells was assessed by measuring vector copy numbers and expression of Wiskott-Aldrich syndrome protein.

**Results:**

After lentiviral vector-mediated GT, the number of transduced B cells progressively increased in the peripheral blood of all patients. Lentiviral vector-transduced progenitor cells were able to repopulate the B-cell compartment with a normal distribution of B-cell subsets both in bone marrow and the periphery, showing a WAS protein expression profile similar to that of healthy donors. In addition, after GT, we observed a normalized frequency of autoimmune-associated CD19^+^CD21^−^CD35^−^ and CD21^low^ B cells and a reduction in B cell–activating factor levels. Immunoglobulin serum levels and autoantibody production improved in all treated patients.

**Conclusions:**

We provide evidence that lentiviral vector-mediated GT induces transgene expression in the B-cell compartment, resulting in ameliorated B-cell development and functionality and contributing to immunologic improvement in patients with WAS.

Wiskott-Aldrich syndrome (WAS; OMIM 301000) is a complex and severe X-linked primary immunodeficiency characterized by both cellular and humoral immunodeficiency, microthrombocytopenia, eczema, and increased risk of autoimmunity and lymphomas.^1,2^ The gene responsible for WAS encodes a 502-amino-acid protein (Wiskott-Aldrich syndrome protein [WASp]) that is a key regulator of actin polymerization.^3^ WASp is specifically expressed in hematopoietic cells^4^ and also exerts important signaling activities independent of cytoskeletal rearrangements.^5-7^ The life expectancy of patients with WAS is severely reduced, unless they are successfully cured by bone marrow (BM) transplantation.^8^ However, a significant fraction of transplanted patients lacks a suitable HLA-matched donor, and patients with a lower degree of chimerism, both in the lymphoid and myeloid compartments, show an incomplete reconstitution of lymphocyte counts and a high incidence of autoimmunity.^8,9^ For these reasons, administration of *WAS* gene–corrected autologous hematopoietic stem cells (HSCs) could represent a valid alternative therapeutic approach, as demonstrated for other primary immunodeficiencies.^10^

In the last 15 years, extensive preclinical studies in human subjects and *Was*^−/−^ mice have evaluated the feasibility and efficacy of *WAS* gene therapy (GT) by means of both retroviral and lentiviral vectors, providing the basis for the clinical application of GT for WAS. The first clinical GT trial for WAS based on gammaretroviral vector-mediated gene transfer, showed sustained expression of WASp in HSCs, lymphoid cells, myeloid cells, and platelets after GT, resulting in a considerable clinical benefit to the patient.^11^ However, the occurrence of leukemias in 7 of 10 treated patients^12^ has raised concerns about the use of non–self-inactivating retroviral vectors.

We developed a GT approach based on a lentiviral vector encoding human WASp cDNA under the control of the human WAS endogenous promoter.^13^ The lentiviral GT protocol is characterized by a reduced-intensity conditioning coupled with depletion of B cells by anti-CD20 antibody administration before the infusion of transduced HSCs. Our initial results in 3 patients showed that lentiviral vector-mediated GT was feasible and led to successful correction of HSCs, resulting in reconstitution of WASp expression in all hematopoietic cell lineages.^14^

Because of the high risk of infections and autoimmune complications, correction of immune cell functions remains the main goal of WAS GT. Both GT trials have described an immunologic improvement about 2 years after treatment in terms of lymphocyte counts, *in vitro* T-cell functionality, use of T-cell receptor Vβ repertoire, natural killer cell immunologic synapse formation, and cytotoxic activity.^11,14^ Several studies on HSC transplantation outcomes demonstrated that successful immune reconstitution and protection from infections require development of humoral immune competence mediated by B lymphocytes.^15^ The B-cell compartment needs to be carefully investigated in the context of GT treatment. Indeed, WAS is characterized by impaired humoral immunity, with skewed immunoglobulin production and defects in polysaccharide antigen response,^2,16^ indicating intrinsic abnormalities of B-cell function. Very recently, B-cell perturbation has been described to contribute to immunodeficiency and autoimmunity in patients with WAS.^17,18^

Thus we evaluated the effects of lentiviral vector-based GT on B-cell homeostasis and distribution, both in the BM and peripheral blood (PB) of patients with WAS until 36 months after treatment. To this end, we followed reconstitution of the B-cell compartment in terms of B-cell counts, B-cell subset distribution, plasma B cell–activating factor (BAFF) and immunoglobulin levels, and autoantibody production in 4 patients enrolled in the lentiviral vector-mediated GT clinical trial.

## Methods

### Patients, treatment, and follow-up

Clinical classification and molecular analysis are described in Table E1 in this article's Online Repository at www.jacionline.org. The clinical protocol (ClinicalTrials.gov, no. NCT01515462) and criteria of eligibility for the study have been previously described.^14^ Human samples were obtained after obtaining informed consent according to the Helsinki Declaration with approval of local medical ethics committees (TIGET02). Some of the samples used as controls have been previously reported.^18^

**Fig E1:**
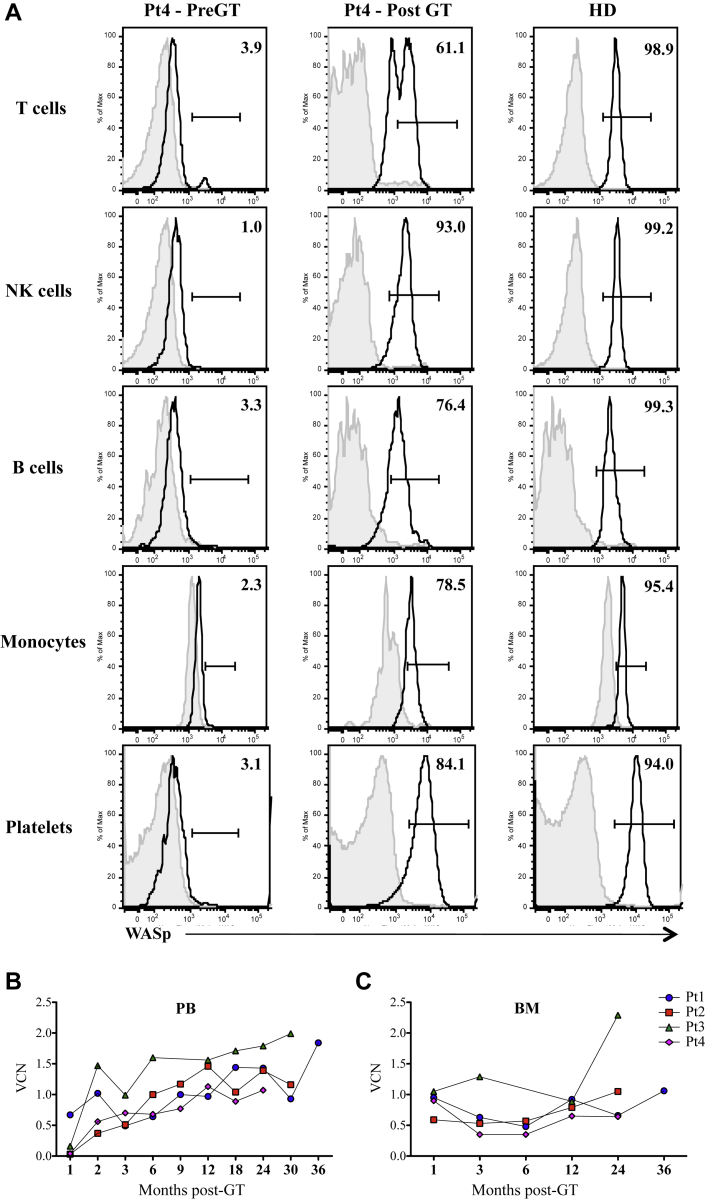
WASp expression and transduction levels after GT. **A,** Histogram plots showing WASp expression measured by means of cytofluorimetric analysis in different hematopoietic cells of Pt4 before and 2 years after GT and an HD. Negative controls are shown as *gray histograms*. **B** and **C,** Transduction levels are shown in the graph as VCNs per genome evaluated by means of quantitative PCR at different time points after GT in B cells purified either from PB (Fig E1, *B*) or BM (Fig E1, *C*).

Four male patients (age range, 1.1-5.9 years) affected by WAS were enrolled for the lentiviral vector-mediated GT and identified as Pt1, Pt2, Pt3, and Pt4. The clinical features of the first 3 patients have been previously described.^14^ Before the treatment, they showed severe clinical conditions, with a Zhu clinical score of 3 to 5 (see Table E1). At the time of treatment, Pt4 was 2.4 years old, with a clinical history of neonatal sepsis; chronic cytomegalovirus infection with frequent reactivations; lymphadenopathies; bleeding manifestations at the skin, gastrointestinal, and brain level; food polyallergy with anaphylaxis; and mild eczema. The patient was given a Zhu score of 5 because of severe refractory thrombocytopenia and the presence of circulating autoantibodies.^19^ All diagnoses were confirmed by genetic analysis and evaluation of WASp expression with flow cytometry (see Table E1). These patients, lacking an HLA-identical sibling donor and matched unrelated donor, underwent GT at a median age of 2.75 years and received a reduced-conditioning regimen protocol preceded by anti-CD20 mAb administration.^14^ CD34^+^ cells isolated from BM in all patients and also from mobilized PB in Pt1^14^ were transduced *ex vivo* with lentiviral w1.6W vector and reinfused into patients at a similar dose (see transduction efficiency and vector copy numbers [VCNs] in Table E1). Pt4, as well as other patients, did not experience mucositis or other chemotherapy-related toxicity. Autoimmune thrombocytopenia persisted in the early follow-up phase in Pt4, and this patient was treated with a high intravenous immunoglobulin (IVIg) dose, anti-CD20 mAb, steroids, and TPO agonists (romiplostim and eltrombopag). Autoantibody levels against platelets became negative 5.1 months after GT, and the patient reached platelet transfusion independence 9 months after GT, with absence of severe bleeding manifestations thereafter. A multilineage expression of WASp was observed in Pt4 after GT, persisting up to the latest time point analyzed (see Fig E1, *A*, in this article's Online Repository at www.jacionline.org).

### Flow cytometric analysis of B-cell subsets and B-cell purification

The composition of the B-cell compartment was analyzed, as previously described.^18^ Briefly, the late stages of B-cell differentiation were analyzed by means of flow cytometric immunophenotyping with the following mAbs: CD34-phycoerythrin (8G12; BD, San Jose, Calif), CD19-PeCy7 (SJ25C1, BD), CD10–fluorescein isothiocyanate (MEM-78; Caltag, Burlingame, Calif), and CD20-phycoerythrin-Texas Red/energy coupled dye (B9E9; Immunotech, Glendale, Calif). B-cell subsets present in PB were characterized by using the following mAbs: CD19-PeCy7 (SJ25C1, BD), CD27-allophycocyanin (M-T271, BD) CD24-PB (SN3; Exbio, Praha, Czech Republic), CD21-allophycocyanin (B-ly4, BD), CD38-PerCP-Cy5.5 (HIT2, BD), and CD35-phycoerythrin (E11, BD). For intracytoplasmic detection of human WASp, cells were fixed and permeabilized with a Cytofix/Cytoperm kit (BD PharMingen). The anti-WASp antibody 503 (a kind gift from Professors H. D. Ochs, Seattle, Wash, and L. D. Notarangelo, Boston, Mass) was used, followed by staining with secondary Alexa Fluor 488– or 647–conjugated goat anti-rabbit antibodies (Invitrogen, Carlsbad, Calif). Samples were acquired on a FACSCanto cytometer and analyzed with FlowJo Software (TreeStar, Ashland, Ore). CD19^+^ cells were purified from mononuclear cells derived from PB or BM by means of positive selection with immunomagnetic beads, according to the manufacturer's procedures (Miltenyi Biotec, Bergisch Gladbach, Germany).

### Chemotaxis assay

The *in vitro* chemotaxis assay was performed with 5 μm/L pore-size Transwell inserts (Costar Corporation, Corning, NY) in 24-well plates, as previously described.^18^ Briefly, CD20^+^ cells were purified from PBMCs of patients with WAS and age-matched healthy donors (HDs) by using immunomagnetic beads (Miltenyi Biotec) and left overnight at 37°C in culture medium. Fifty thousand CD20^+^ cells were seeded in the upper chamber. In the bottom well we placed culture medium supplemented with 250 ng/mL recombinant human stromal cell–derived factor 1α (SDF-1α; CXCL12; PeproTech, Rocky Hill, NJ) or medium alone and incubated at 37°C for 3 hours. Cells migrated into the lower chamber were counted for viable cells and stained with anti-CD19, anti-CD24, anti-CD38, and anti-CD27 antibodies (BD Biosciences) for phenotypic analysis by using fluorescence-activated cell sorting. Migration frequency was estimated as the percentage of migrated cells over the input cell number.

### VCN

The number of lentiviral vector copies integrated per genome was evaluated by using quantitative PCR, as previously described.^20^ Results of integrated vector copies were normalized for the number of evaluated genomes. All the reactions were performed according to the manufacturer's instructions and analyzed with an ABI PRISM 7900 sequence detection system (Applied Biosystems, Foster City, Calif).

### ELISA

BAFF levels were measured in duplicate in plasma samples from patients with WAS and HDs by using a Quantikine Human BAFF/BLyS/TNFSF13B Immunoassay kit (R&D Systems, Minneapolis, Minn). The assay was performed according to the manufacturer's instructions, and OD values were evaluated at 450 nm.

### Evaluation of immunoglobulin levels and presence of autoantibodies

Immunoglobulin levels and presence of autoantibodies were determined by Laboraf Diagnostics and Research (San Raffaele Hospital, Milan, Italy), according to internal standard procedures. Antibodies against platelets were detected by using a commercially available method, the solid phase red cell adherence test (SPRCA test, P-Capture Ready Screen; Immucor, Norcross, Ga), which was designed for laboratory detection of IgG anti-platelet antibodies.^21-23^

Plasma samples isolated from all patients with WAS before and 2 years after GT were also screened for the presence of autoantibodies by using an autoantigen proteomic microarray comprising 123 different antigens. Autoantigen microarrays were manufactured, hybridized, and scanned by the Microarray Core Facility at the University of Texas Southwestern Medical Center (Dallas, Tex)^24^ in a blind manner. A heat map was generated based on the normalized fluorescent intensity of autoantibodies and on a color scale range between +2 and −2 SDs. Values were mean centered, and antigens with a signal-to-noise ratio of less than 3 were considered undetectable and removed in both pre-GT and post-GT samples, thus reducing the number of antigens from 123 to 93. Differences related to each antigen between pre- and post-GT samples were evaluated by using the paired *t* test, and a global test *P* value was computed by using the global test R package to evaluate differences related to the whole antigen profile between pre- and post-GT samples.

### Statistical analysis

All results are expressed as means ± SDs, if not stated otherwise. Statistical significance was assessed by using 2-tailed Mann-Whitney tests for comparisons between patients and HDs or paired *t* tests for comparisons before and after treatment. *P* values of less than .05 were considered significant.

## Results

### Treatment and follow-up

After treatment with lentiviral vector-mediated GT, all patients showed a stable and multilineage engraftment of transduced HSCs, with expression of WASp progressively increasing and persisting in the different hematopoietic cell lineages. No adverse events related to the treatment were observed after GT, and all patients were alive and clinically well at the time of this study (Aiuti et al^14^ and unpublished results). Frequency and severity of infections and bleeding were reduced after the first year of follow-up. All patients became independent from platelet transfusions, with platelet counts progressively increasing over time, although not reaching normal levels. Eczema improved in all 4 patients, and no clinical manifestations of autoimmunity were observed after the first year of follow-up. All patients discontinued anti-infectious prophylaxis, and 2 of 4 (Pt1 and Pt3) stopped IVIg supplementation as well. After IVIg suspension, the 2 patients received regular vaccinations, and both showed a protective response to T cell–dependent antigens. Pt1 produced specific IgM after 2 doses of unconjugated vaccine (Pneumovax; Merck & Co, Whitehouse Station, NJ). Because protective antibody levels were not reached, the patient received 2 doses of conjugated pneumococcal vaccine (Prevnar 13; Wyeth, Dallas, Tex) and was able to mount a protective response.

### Reconstitution and gene correction of B lymphocytes

**Fig 1:**
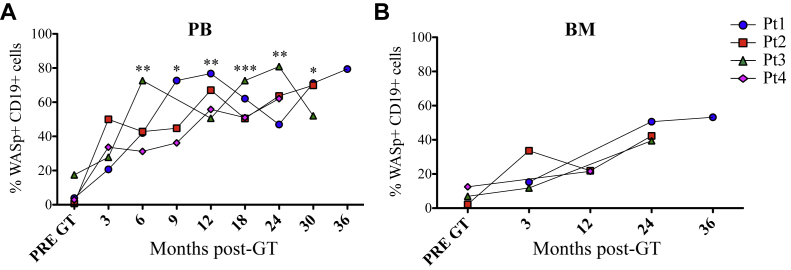
WASp expression in B cells after GT. WASp-expressing B cells in PB **(A)** and BM **(B)**. *Asterisks* define the difference between pre-GT and post-GT samples. ***P ≤ .0005, **P ≤ .005, and *P < .05.

**Fig 2:**
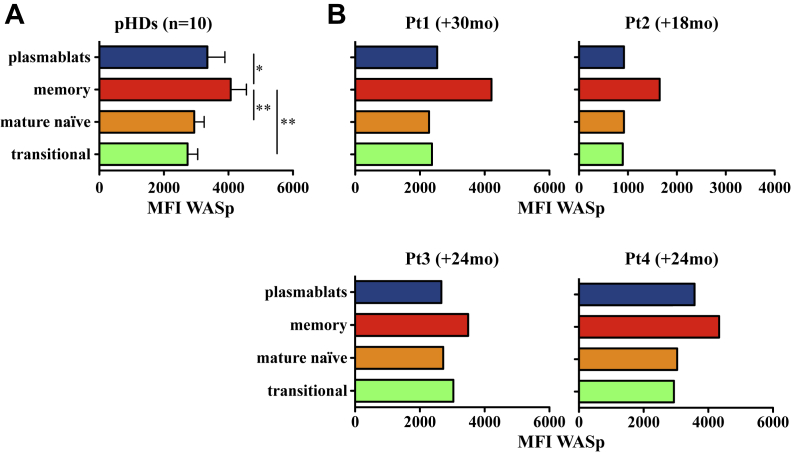
Expression of WASp among B-cell populations. **A,** WASp mean fluorescence intensity *(MFI)* in PB B-cell subsets of pediatric HDs *(pHDs)*. *Bars* indicate means and SEMs. **P < .005 and *P < .05. **B,** WASp MFI in B-cell subsets of treated patients is reported in individual graphs, with the time of the analysis indicated as months after GT between parentheses. All MFI values are normalized to the MFI of the negative control stained only with secondary antibody.

We have previously demonstrated the efficacy of lentiviral w1.6W vector in restoring B-cell defects in a WAS mouse model.^25^ On the basis of this evidence, we evaluated the effect of GT on the B-cell compartment in patients with WAS. To this end, we followed the reconstitution, transduction levels, and expression of WASp in B lymphocytes of 4 patients with WAS treated with GT. Before treatment, only Pt1 presented with few CD19^+^ B cells in PB, whereas B-cell counts were in the normal range in the remaining patients (Aiuti et al^14^ and Table I).^14,26^ During the first months after GT treatment, we observed a low number of B cells (unpublished results), as expected because of the combined effect of conditioning regimen and anti-CD20 mAb administration, which progressively increased, reaching the range of normality (Table I). All treated patients expressed WASp in CD19^+^ cells (Figs 1 and 2),^14^ demonstrating that newly generated B cells arising from transduced progenitor cells stably expressed the transgene. In PB the frequency of CD19^+^ cells expressing WASp was significantly increased 6 months after GT and remained stable over time at the last follow-up (Fig 1, *A*).

When feasible, we analyzed WASp expression in BM-isolated B cells in parallel to PB analysis (Fig 1, *B*). Higher frequencies of WASp-positive B cells were detected in PB compared with BM in all patients (Fig 1). Transduction levels were evaluated as VCNs in B cells isolated from PB and BM (see Fig E1, *B* and *C*). As previously shown,^14^ proportion of transduced B cells progressively increased in PB of all patients from the first months after GT. Furthermore, the higher WASp expression level in PB is in line with the higher VCN found in PB compared with BM, indicating a selective advantage of gene-corrected B cells in the periphery.

### Kinetics of B-cell reconstitution in BM and PB after GT

**Fig 3:**
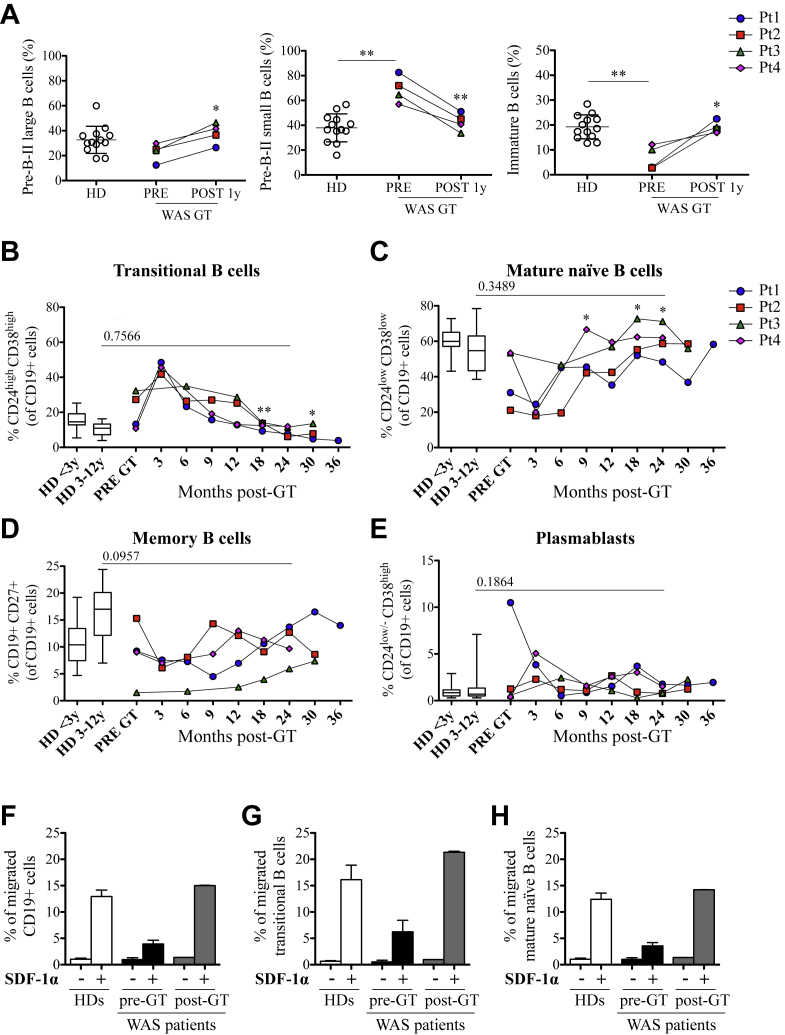
B-cell differentiation and *in vitro* migratory ability of B cells after GT. **A,** Proportion of pre-B-II large (CD10^int^CD20^int^), pre-B-II small (CD10^int^CD20^−^), and immature B cells (CD10^int^CD20^+^) determined on CD34^−^CD19^+^ cells and calculated on total BM precursor B cells, excluding recirculating mature B cells (CD10^−^CD20^+^). **B-E,** Proportion of B-cell subsets in PB (gated on CD19^+^; HD <3 years, n = 26; HD 3-12 years, n = 20). *Box plots* show medians, 25th and 75th percentiles, minimums, and maximums. *Asterisks above horizontal lines* indicate significant differences between patients with WAS and HDs. *Asterisks without lines* define differences between pre-GT and post-GT samples. **P ≤ .005 and *P < .05. **F-H,***In vitro* migration of CD20^+^ cells isolated from age-matched HDs (*white bars*, n = 10) and patients with WAS before *(black bars)* and after *(gray bars)* GT (n = 2, Pt2 and Pt3) in the presence of SDF-1α *(+)* or medium alone *(−)*. Percentages of migrated CD19^+^ cells (Fig 3, *F*), transitional cells (Fig 3, *G*), and mature naive B cells (Fig 3, *H*) were determined by using flow cytometric analysis.

We have previously demonstrated that WASp deficiency perturbs B-cell development, starting in the BM, where we described an altered distribution of B-cell precursor subsets.^18^ Thus we evaluated whether GT treatment could restore a normal distribution pattern in BM. As expected, patients showed a significantly high proportion of small pre-B-II cells (CD34^−^CD19^+^CD10^int^CD20^−^) and a low proportion of immature B cells (CD34^−^CD19^+^CD10^int^CD20^+^) before GT, which reverted to normal in all patients 1 year after treatment (Fig 3, *A*).

Next, we evaluated PB B-cell subpopulation distribution before and after treatment. Before GT, transitional B-cell frequencies (CD19^+^CD24^high^CD38^high^) were higher in Pt2 and Pt3 when compared with HDs (Fig 3, *B*). Three months after treatment, transitional B cells represented the first subset detected at a high proportion in PB and then progressively decreased to proportions lower than pre-GT values (at 30 months after GT, *P* = .049), reaching normal values 24 months after GT (*P* = .7566; Fig 3, *B*). In parallel, the proportion of mature naive B cells (CD19^+^CD24^dim^CD38^dim^) increased over time to reach values comparable with those observed in HDs (*P* = .3489; Fig 3, *C*). With regard to mature B-cell subsets, we observed that Pt1, Pt2, and Pt4 had normal percentages of memory B cells (CD19^+^CD27^+^) before and after GT (Fig 3, *D*). Pt3 displayed a severe reduction in memory B-cell frequency before GT (1.5% WAS before GT vs 4.7% to 19.2%, minimum and maximum values of HDs at <3 years, respectively), which increased up to values within the HD range 30 months after treatment (7.38% Pt3 vs 7.0% to 24.4%, minimum and maximum values of HDs at 3-12 years, respectively; Fig 3, *D*). Analysis of switched memory B cells in treated patients showed a trend similar to that of total memory B cells 2 years after GT (unpublished results). Plasmablasts (CD19^+^CD24^−^CD38^high^) were in the normal range of values before and after GT, with the exception of Pt1, who presented with a higher plasmablast proportion before GT that normalized after therapy (Fig 3, *E*).

To evaluate whether the normalization of B-cell subset distribution in the BM and PB in treated patients was associated with a qualitative improvement in B-cell function, we tested their *in vitro* chemotactic response to SDF-1α, which we previously described as defective in patients with WAS.^18^ We confirmed that WASp-deficient B cells isolated from 2 patients (Pt2 and Pt3) before GT were less responsive to SDF-1α and observed that they had a normal migratory ability after GT (Fig 3, *F-H*). Indeed, the percentages of *in vitro*–migrated transitional (Fig 3, *G*) and mature naive (Fig 3, *H*) B cells after GT were higher than those before GT and comparable with those of HDs.

Because the absence of WASp perturbs B-cell subset distribution in human subjects^17,18,27^ and mice,^28,29^ we compared differential WASp expression during B-cell maturation in HDs and patients with WAS after GT. When we evaluated the mean fluorescence intensity levels of WASp in different B-cell subsets in the blood of pediatric HDs, we detected a differential pattern of WASp expression among peripheral B-cell subsets. WASp is expressed at significantly higher levels in memory B cells compared with levels in transitional and mature naive B cells (Fig 2, *A*), suggesting that WASp expression is modulated in the different B-cell populations. In patients undergoing GT, we noticed the same pattern of WASp expression in different B-cell subpopulations, in particular in memory B cells (Fig 2, *B*). These data strongly suggest that the lentiviral vector-transduced CD34^+^ cells were able to repopulate the B-cell compartment with a normal distribution of B-cell subsets both in BM and the periphery, presenting a WASp expression profile similar to that of HDs.

### Correction of immunophenotypic perturbation of B cells from patients with WAS after GT

To further characterize the composition of the peripheral B-cell compartment, we analyzed the expression of 2 complement receptors, CD35 and CD21, involved in maintenance of self-tolerance^30,31^ and less expressed by B cells from patients with WAS.^18,27^

**Fig 4:**
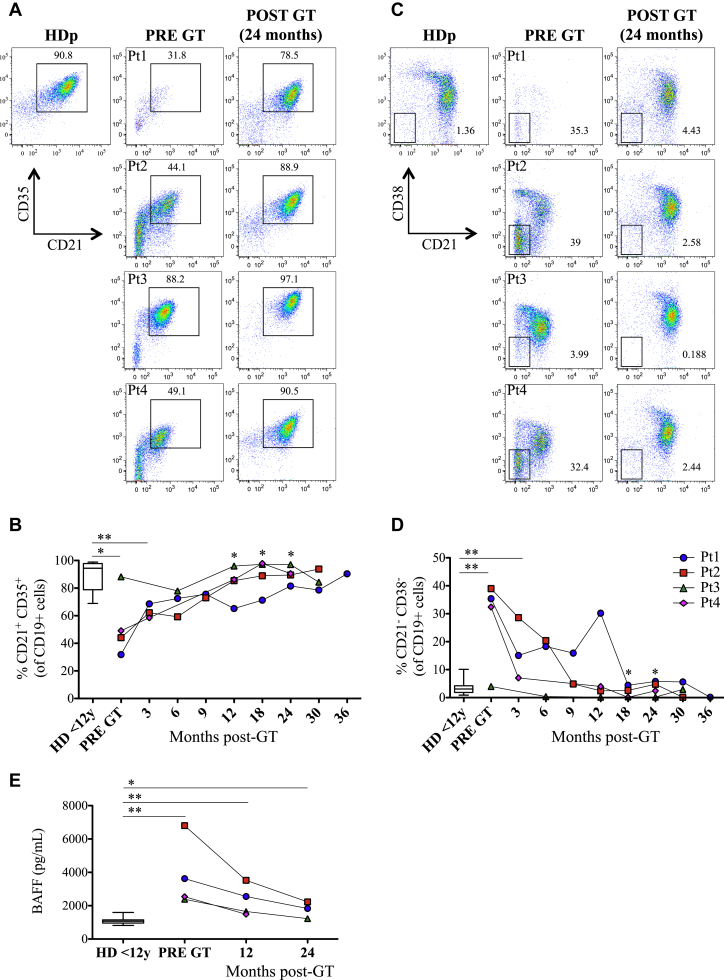
Normalization of phenotypic alterations of B cells from patients with WAS and BAFF levels after GT. **A** and **B,** CD21 and CD35 expression on CD19^+^ cells in patients with WAS before and after GT and in HDs (n = 16). **C** and **D,** CD21^low^ B cells in patients with WAS before and after GT and in HDs (n = 45). **E,** BAFF levels before and after GT and in HDs (n = 24). *Asterisks above horizontal lines* indicate significant differences between patients with WAS and HDs. *Asterisks without lines* define differences between pre-GT and post-GT samples. **P ≤ .005 and *P < .05.

When we analyzed the expression of CD21 and CD35 before GT, we found a lower proportion of double-positive cells in all patients compared with HDs, with the exception of Pt3 (Fig 4, *A* and *B*). Three months after GT, an increase in the percentage of CD19^+^CD21^+^CD35^+^ B cells was already noticeable and reached statistical significance 1 year after GT compared with values before treatment (mean frequency of all patients 1 year after GT is 85.8% compared with 46.6% before GT). Moreover, the frequency of CD19^+^CD21^+^CD35^+^ B cells remained stable until the last follow-up, with levels similar to those of HDs (Fig 4, *B*). Interestingly, Pt3 showed complete absence of the CD21/CD35 double-negative population 2 years after treatment.

The B-cell compartment of patients with WAS is also characterized by the presence of an unusual population phenotypically identified as CD19^+^CD21^−^CD38^−^ (referred to as CD21^low^)^18^ that were enriched in autoreactive and unresponsive B cells that could be involved in the breakdown of B-cell tolerance.^32-34^ Before treatment, patients with WAS presented with an increased frequency of CD21^low^ B cells, which diminished 3 months after GT, becoming nearly absent starting at 18 months after treatment (Fig 4, *C* and *D*). We also assessed plasma BAFF levels, which are known to play a role in B-cell homeostasis and peripheral tolerance.^35,36^ Higher BAFF levels were found in all patients before GT compared with those of age-matched HDs (*P* = .0018; Fig 4, *E*). GT treatment led to a decrease in BAFF plasma levels in all treated patients, although they still remained statistically different from HDs (.0018 vs .015, *P* value of pre-GT patients and HDs vs *P* value of patients with WAS 30 months after GT and HDs).

### *In vivo* B-cell function after GT

To evaluate B-cell functionality, we monitored production of immunoglobulins and autoantibodies before and after GT. Pt1^14^ and Pt3 have been taken off IVIg administration 10 and 20 months after treatment, respectively. Pt3 had IgG serum levels within the normal range at the last determination point, whereas in Pt1 IgG serum levels were less than the normal range (Table II). The evaluation of IgG levels in Pt2^14^ and Pt4 was not informative because of IVIg administration. Of note, Pt2 and Pt4 experienced autoimmune thrombocytopenia in the first months after GT and received high doses of IVIg and anti-CD20 mAb, which might have slowed down the reconstitution of the B-cell compartment and delayed IVIg discontinuation. Before GT, patients showed variable levels of immunoglobulin classes. Pt1 had low IgM serum levels, which improved after treatment but remained less than normal levels. IgE levels improved in Pt2 after treatment, although IgM and IgA levels were still out of the range of normality. Pt3 showed higher IgA and IgE serum levels before treatment, which decreased after therapy. Pt4 showed lower IgM and higher IgE levels compared with HDs but improved after GT (Table II).

Patients with WAS have a higher risk of autoimmune manifestations,^2^ even after HSC transplantation, when complete chimerism is not achieved.^8^ Thus we examined the production of autoantibodies in the sera of patients with WAS undergoing GT before and after GT (Table III). No positivity for anti–liver kidney microsomal antibodies, anti–smooth muscle antibodies, anti-mitochondrial antibodies, anti-DNA, perinuclear ant-neutrophil cytoplasmic antibodies, anti-neutrophil cytoplasmic antibodies, and direct and indirect Coombs tests was found before and after GT. Before GT, Pt3 showed anti-platelet antibodies, which were still present 2 years after GT, whereas anti-nuclear antibody levels normalized after GT. In Pt4 anti-platelet antibodies were present before treatment and disappeared at the last follow-up.

**Fig 5:**
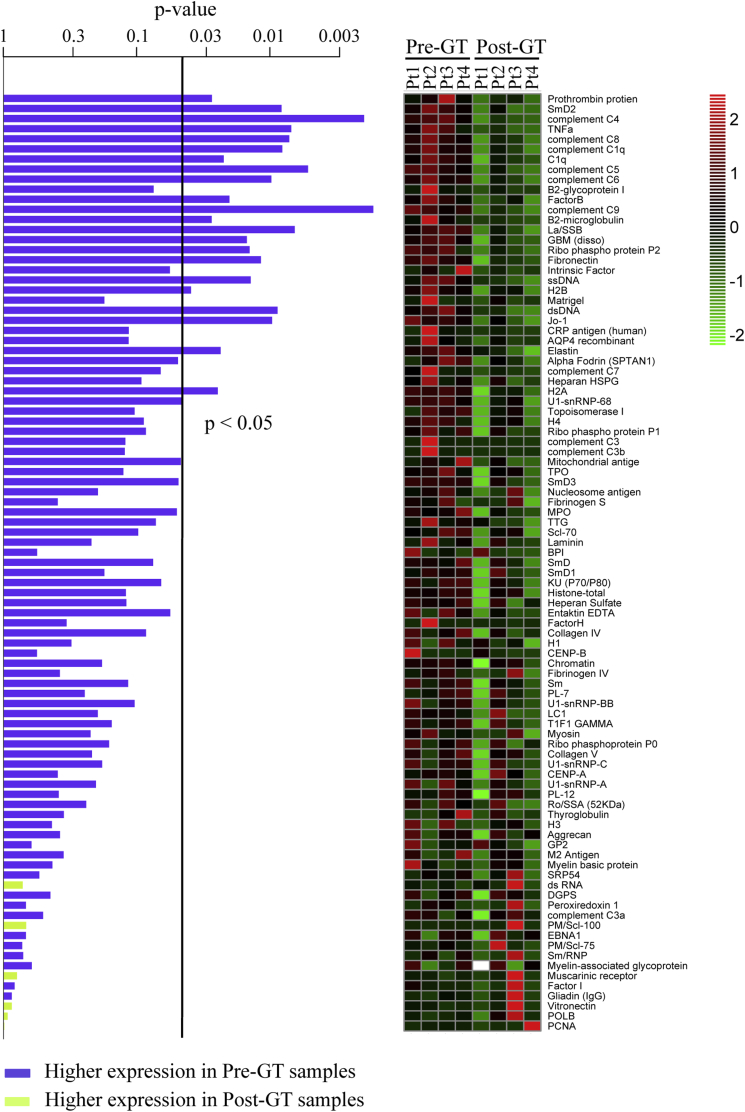
Decreased autoantibody production in patients with WAS after GT. IgG reactivities against 123 antigens were tested by using an autoantigen array. *P* values resulting from paired *t* tests of differential expression between pre-GT and matched post-GT samples are shown in the *left panel*. *Blue* and *green bars* refer to autoantibodies with higher expression in pre-GT and post-GT samples, respectively. The *vertical line* indicates a *P* value of less than .05. Data are represented as a heat map *(right panel)*, with values mean centered and colors scaled from −2 to +2 SD. The legend shows *z* scores.

Furthermore, to extend the analysis to a broader panel of autoantigens, we used a high-throughput autoantigen microarray platform that contains 123 different autoantigens. We observed a trend toward global decreased reactivity to autoantigens in the plasma of patients with WAS 2 years after therapy versus before GT (global test *P* = .0693). Several antigens were significantly downregulated (*P* < .05, Fig 5).

## Discussion

In the present study we demonstrate that lentiviral vector-transduced hematopoietic progenitor cells can differentiate into WASp-expressing B cells, leading to normalization of B-cell development both in BM and PB. We show the correction of phenotypic perturbations of B cells from patients with WAS and a decrease in plasma BAFF concentrations toward levels in age-matched HDs. In addition, B-cell reconstitution led to discontinuation of IVIg infusions in 2 patients (Pt1 and Pt3).

We observed that GT normalized the proportions of early B-cell subsets, indicating that B-cell development occurs normally in the BM after GT. An increased proportion of immature B cells was observed in the BM of treated patients in association with a decrease in transitional B-cell numbers in the periphery in contrast to what has been described in untreated patients with WAS.^18^ We have previously demonstrated that the overrepresentation of transitional B cells in the PB of patients with WAS is, at least in part, due to an early export of immature B cells from the BM because of the inability of B cells from patients with WAS to sense retention signals mediated by CXCR4 and its ligand, SDF-1α.^18^ Thus the restoration of proper retention signals mediated by WASp could contribute to the normalization of immature B cells in the BM and transitional B cells in the periphery.

Several studies in WAS murine models have shown that WASp is crucial for peripheral B-cell homeostasis but could be dispensable during early murine hematopoiesis.^25,28,29,37^ Our analysis of WASp expression in B cells from patients undergoing GT confirms the selective advantage of WASp-expressing cells in the periphery with respect to the BM compartment. In particular, at the peripheral level WASp is highly expressed in mature B cells, such as memory cells and plasmablasts. These data are in agreement with findings reporting a selective advantage of WASp-positive mature B cells in competitive transplant settings and heterozygous mice^28,29^ and further confirm the preclinical data of lentiviral vector-mediated GT in *Was*^−/−^ mice.^25^ However, similar to the retroviral vector-mediated GT trial,^11^ WASp-negative cells are still present in the B-cell compartment in our patients. This is in line with results obtained from preclinical studies conducted in sublethally irradiated *Was*^−/−^ mice treated with *Was*^−/−^ cells transduced with the w1.6W lentiviral vector (the same vector used in our trial). However, even in the presence of mixed chimerism of WASp-positive and WASp-negative B cells, GT led to an improvement in B-cell function in terms of response to T cell–independent antigens and autoantibody titers.^25^ In our cohort of patients with WAS, 2 of 4 treated patients discontinued immunoglobulin treatment, and 1 is decreasing the frequency of infusions, suggesting that *in vivo* B-cell function is being restored.

Importantly, the reduced-intensity conditioning regimen (low doses of busulfan and fludarabine) and administration of anti-CD20 mAb, which is chosen in patients undergoing GT to avoid conditioning-related toxicity, might affect the kinetics of B-cell reconstitution. Indeed, observations obtained from the outcome of HSC transplantation in a cohort of pediatric patients with WAS indicate a better disease-free survival in patients receiving a fully myeloablative conditioning regimen,^38^ similar to observations previously reported for B-cell reconstitution in patients with severe combined immunodeficiencies.^39^ To this end, data from ongoing GT clinical trials performed in different centers using the same clinical vector in patients with WAS but different conditioning regimens will be instrumental to understand the relevance of conditioning in the kinetics of immune reconstitution in autologous BM transplantation.

During B-cell development, the maintenance of the primary B-cell pool and the fate of self-reactive B cells are largely coordinated by B-cell receptor, BAFF, and its receptor.^35,40^ Before GT, increased BAFF plasma levels, which might decrease the threshold for the survival of autoreactive B-cell clones,^35^ were found in patients with WAS similar to those reported in patients with other immunodeficiencies^41^ and autoimmune diseases.^42-45^ Thus we hypothesize that the reduced BAFF plasma levels found in GT-treated patients, reflecting the improvement in B-cell lymphopenia and B-cell subset distribution,^41^ could ultimately result in a less favorable environment for survival of autoreactive B cells. Consistently, we observed a significant decrease in CD21^low^ B-cell frequencies, which were described to be enriched in anergic autoreactive clones^32^ and expanded in patients with autoimmune diseases.^32,34,46^ CD21^low^ B cells, which were probably removed by anti-CD20 administration, might not find favorable conditions to expand after GT treatment. However, patients have not yet normalized BAFF levels 2 years after GT, suggesting that full recovery of the B-cell compartment might require more time. Of note, the slow kinetics of BAFF level reduction were also observed in adenosine deaminase–deficient patients after GT^47^ and might be associated with the use of a reduced-intensity conditioning.^48^ Finally, upon GT treatment, we observed a correction of the expression of the complement receptors CD21 and CD35, which are involved in the capture and presentation of opsonized antigens^49^ and in negative selection of self-reactive B lymphocytes^30^ and represent a common trait in patients with WAS.^18,27^

In conclusion, although longer follow-up is required to evaluate full B-cell restoration, our data indicate that the lentiviral vector-mediated GT treatment for WAS is efficacious in inducing WASp expression in B cells, leading to a correction of phenotypic perturbation and resulting in a robust and stable B-cell reconstitution.

**Clinical implications**

Lentiviral vector-mediated GT treatment for WAS restores WASp expression in B cells and corrects phenotypic perturbations.

## Figures and Tables

**Table E1:** Features of patients with WAS treated with lentiviral vector-mediated GT

	Pt1∗	Pt2∗	Pt3∗	Pt4
Zhu score	3	4	4	5
*WAS* mutation	Exon 10 c.961C>T (R321X)	Exon 10 c.1337_1338+9del	Exon 1 c.37C>T (R13X)	Exon 1 c.91G>A (E31K)
WASp expression	<5%	<5%	<5%	<5%
Years at treatment	5.9	1.6	1.1	2.4
Cell dose (×10^6^/kg)	3.66 (BM) + 5.25 (MPB)	14.1 (BM)	10.2 (BM)	10.3 (BM)
VCN	1.9-1.4	2.4	2.8	2.4
Transduction efficiency in CFC	92% (BM); 88% (MPB)	97%	100%	94%
FU at study analysis	36 mo	30 mo	30 mo	24 mo
Current clinical condition	Alive and well No eczema No major bleeding Off IVIg	Alive and well No eczema No major bleeding Receiving IVIg	Alive and well No eczema No major bleeding Off IVIg	Alive and well No eczema No severe infections Receiving IVIg

*c.*, Coding DNA; *CFC*, colony-forming cells; *FU*, follow-up; *MPB*, mobilized peripheral blood.

∗ Aiuti et al.^14^

**Table I:** Absolute B-cell counts in the blood of patients with WAS before and after GT

	Pt1		Pt2		Pt3		Pt4	
Before GT	+36 mo	Before GT	+30 mo	Before GT	+30 mo	Before GT	+24 mo
CD19 (10^9^/L)	**0.125**∗ (0.2-1.6)	0.333 (0.2-1.6)	0.617∗ (0.6-3.1)	0.257 (0.2-2.1)	0.742∗ (0.6-2.7)	1.895 (0.2-2.1)	0.333 (0.2-2.1)	0.240 (0.2-2.1)

Absolute counts are shown before GT and at the last follow-up after GT for all patients. Reference values for the 5th and 95th percentiles of the respective age-matched HDs were reported by Comans-Bitter et al^26^ and are indicated in parentheses. Values out of the normal range are shown in boldface.

∗ Values previously published.^14^

**Table II:** Immunoglobulin levels in the sera of patients with WAS before and after GT

	Pt1		Pt2		Pt3		Pt4	
Before GT	+36 mo	Before GT	+30 mo	Before GT	+30 mo	Before GT	+24 mo
IgG (g/L)	5.21 (3.4-12.4)	**5.45** (6.5-16.0)	7.93∗ (3.4-12.4)	6.68∗ (3.4-12.4)	**14.01**∗ (3.4-12.4)	8.95 (3.4-12.4)	9.29∗ (3.4-12.4)	4.95∗ (3.4-12.4)
IgM (g/L)	**0.11** (0.48-2.2)	**0.38** (0.48-2.2)	0.77 (0.48-2.2)	**0.35** (0.48-2.2)	0.59 (0.48-2.2)	0.68 (0.48-2.2)	**0.28** (0.48-2.2)	**0.44** (0.48-2.2)
IgA (g/L)	1.51 (0.35-2.0)	1.38 (0.45-2.5)	**1.63** (0.15-1.1)	**1.99** (0.18-1.6)	**4.18** (0.15-1.1)	**4.69** (0.18-1.6)	0.8 (0.18-1.6)	0.58 (0.18-1.6)
IgE (UI/mL)	24 (1.0-60.0)	30.8 (1.0-60.0)	**184** (1.0-60.0)	55 (1.0-60.0)	**314** (1.0-60.0)	**97** (1.0-60.0)	**180** (1.0-60.0)	**117** (1.0-60.0)

Serum IgG, IgM, IgA, and IgE levels before GT and at the last follow-up are shown. The corresponding interquartile ranges of age-matched HDs (Laboraf reference values) are reported in parentheses. Values out of the normal range are shown in boldface.

∗ Patient receiving IVIg therapy.

**Table III:** Autoantibodies in the sera of patients with WAS before and after GT

	LKM	ASMA	AMA	Anti-DNA	p-ANCA	c-ANCA	Direct Coombs	Indirect Coombs	Anti-plt	ANA∗	∗if + search for ENA
Pt1											
Before GT	−	−	−	−	−	−	−	−	−	−	ND
+1 y	−	−	−	−	−	−	ND	ND	−	−	ND
+2 y	−	−	−	−	−	−	−	−	ND	−	ND
Pt2											
Before GT	−	−	−	−	−	−	−	−	−	−	−
+1 y	−	−	−	−	−	−	−	−	−	−	−
+2 y	−	−	−	−	−	−	−	−	−	−	−
Pt3											
Before GT	−	−	−	−	−	−	−	−	+	+1:160	−
+1 y	−	−	−	−	−	−	−	−	ND	−	−
+2 y	−	−	−	−	−	−	−	−	+	−	−
Pt4											
Before GT	−	−	−	−	−	−	−	−	+	−	−
+1 y	−	−	−	−	−	−	−	−	−	−	−
+2 y	−	−	−	−	−	−	−	−	−	−	ND

*−*, Negativity to antigens; *+*, positivity to antigens together with autoantibody titers; *AMA*, anti-mitochondrial antibodies; *ANA*, anti-nuclear antibodies; *anti-plt*, anti-platelet antibodies; *ASMA*, anti–smooth muscle antibodies; *c-ANCA*, cytoplasmic anti-neutrophil cytoplasmic antibodies; *ENA*, anti-extractable nuclear antibodies; *LKM*, anti–liver kidney microsomal antibodies; *ND*, not done; *p-ANCA*, perinuclear anti-neutrophil cytoplasmic antibodies.

∗ Patient receiving IVIg therapy.

## References

[1] Bosticardo M., Marangoni F., Aiuti A., Villa A., Grazia Roncarolo M. (2009). Recent advances in understanding the pathophysiology of Wiskott-Aldrich syndrome. Blood.

[2] Catucci M., Castiello M.C., Pala F., Bosticardo M., Villa A. (2012). Autoimmunity in wiskott-Aldrich syndrome: an unsolved enigma. Front Immunol.

[3] Symons M., Derry J.M., Karlak B., Jiang S., Lemahieu V., Mccormick F. (1996). Wiskott-Aldrich syndrome protein, a novel effector for the GTPase CDC42Hs, is implicated in actin polymerization. Cell.

[4] Stewart D.M., Treiber-Held S., Kurman C.C., Facchetti F., Notarangelo L.D., Nelson D.L. (1996). Studies of the expression of the Wiskott-Aldrich syndrome protein. J Clin Invest.

[5] Huang W., Ochs H.D., Dupont B., Vyas Y.M. (2005). The Wiskott-Aldrich syndrome protein regulates nuclear translocation of NFAT2 and NF-kappa B (RelA) independently of its role in filamentous actin polymerization and actin cytoskeletal rearrangement. J Immunol.

[6] Silvin C., Belisle B., Abo A. (2001). A role for Wiskott-Aldrich syndrome protein in T-cell receptor-mediated transcriptional activation independent of actin polymerization. J Biol Chem.

[7] Taylor M.D., Sadhukhan S., Kottangada P., Ramgopal A., Sarkar K., D’Silva S. (2010). Nuclear role of WASp in the pathogenesis of dysregulated TH1 immunity in human Wiskott-Aldrich syndrome. Sci Transl Med.

[8] Moratto D., Giliani S., Bonfim C., Mazzolari E., Fischer A., Ochs H.D. (2011). Long-term outcome and lineage-specific chimerism in 194 patients with Wiskott-Aldrich syndrome treated by hematopoietic cell transplantation in the period 1980-2009: an international collaborative study. Blood.

[9] Ozsahin H., Cavazzana-Calvo M., Notarangelo L.D., Schulz A., Thrasher A.J., Mazzolari E. (2008). Long-term outcome following hematopoietic stem-cell transplantation in Wiskott-Aldrich syndrome: collaborative study of the European Society for Immunodeficiencies and European Group for Blood and Marrow Transplantation. Blood.

[10] Candotti F. (2014). Gene transfer into hematopoietic stem cells as treatment for primary immunodeficiency diseases. Int J Hematol.

[11] Boztug K., Schmidt M., Schwarzer A., Banerjee P.P., Díez I.A., Dewey R.A. (2010). Stem-cell gene therapy for the Wiskott-Aldrich syndrome. N Engl J Med.

[12] Braun C.J., Boztug K., Paruzynski A., Witzel M., Schwarzer A., Rothe M. (2014). Gene therapy for Wiskott-Aldrich syndrome—long-term efficacy and genotoxicity. Sci Transl Med.

[13] Dupré L., Trifari S., Follenzi A., Marangoni F., Lain de Lera T., Bernad A. (2004). Lentiviral vector-mediated gene transfer in T cells from Wiskott-Aldrich syndrome patients leads to functional correction. Mol Ther J Am Soc Gene Ther.

[14] Aiuti A., Biasco L., Scaramuzza S., Ferrua F., Cicalese M.P., Baricordi C. (2013). Lentiviral hematopoietic stem cell gene therapy in patients with Wiskott-Aldrich syndrome. Science.

[15] Small T.N., Robinson W.H., Miklos D.B. (2009). B cells and transplantation: an educational resource. Biol Blood Marrow Transplant.

[16] Ochs H.D., Slichter S.J., Harker L.A., Von Behrens W.E., Clark R.A., Wedgwood R.J. (1980). The Wiskott-Aldrich syndrome: studies of lymphocytes, granulocytes, and platelets. Blood.

[17] Simon K.L., Anderson S.M., Garabedian E.K., Moratto D., Sokolic R.A., Candotti F. (2014). Molecular and phenotypic abnormalities of B lymphocytes in patients with Wiskott-Aldrich syndrome. J Allergy Clin Immunol.

[18] Castiello M.C., Bosticardo M., Pala F., Catucci M., Chamberlain N., van Zelm M.C. (2014). Wiskott-Aldrich Syndrome protein deficiency perturbs the homeostasis of B-cell compartment in humans. J Autoimmun.

[19] Mahlaoui N., Pellier I., Mignot C., Jais J.-P., Bilhou-Nabéra C., Moshous D. (2013). Characteristics and outcome of early-onset, severe forms of Wiskott-Aldrich syndrome. Blood.

[20] Scaramuzza S., Biasco L., Ripamonti A., Castiello M.C., Loperfido M., Draghici E. (2013). Preclinical safety and efficacy of human CD34(+) cells transduced with lentiviral vector for the treatment of Wiskott-Aldrich syndrome. Mol Ther.

[21] Shibata Y., Juji T., Nishizawa Y., Sakamoto H., Ozawa N. (1981). Detection of platelet antibodies by a newly developed mixed agglutination with platelets. Vox Sang.

[22] Rachel J.M., Sinor L.T., Tawfik O.W., Summers T.C., Beck M.L., Bayer W.L. (1985). A solid-phase red cell adherence test for platelet cross-matching. Med Lab Sci.

[23] Jones C.D., Gould L.M., Lee S. (1990). An evaluation of a solid phase red cell adherence test for detecting platelet-associated IgG in immune thrombocytopenia. Am J Clin Pathol.

[24] Li Q.-Z., Zhou J., Wandstrat A.E., Carr-Johnson F., Branch V., Karp D.R. (2007). Protein array autoantibody profiles for insights into systemic lupus erythematosus and incomplete lupus syndromes. Clin Exp Immunol.

[25] Bosticardo M., Draghici E., Schena F., Sauer A.V., Fontana E., Castiello M.C. (2011). Lentiviral-mediated gene therapy leads to improvement of B-cell functionality in a murine model of Wiskott-Aldrich syndrome. J Allergy Clin Immunol.

[26] Comans-Bitter W.M., de Groot R., van den Beemd R., Neijens H.J., Hop W.C., Groeneveld K. (1997). Immunophenotyping of blood lymphocytes in childhood. Reference values for lymphocyte subpopulations. J Pediatr.

[27] Park J.Y., Shcherbina A. (2005). Phenotypic perturbation of B cells in the Wiskott-Aldrich syndrome. Clin Exp Immunol.

[28] Westerberg L.S., de la Fuente M., Wermeling F., Ochs H.D., Karlsson M.C.I., Snapper S.B. (2008). WASP confers selective advantage for specific hematopoietic cell populations and serves a unique role in marginal zone B-cell homeostasis and function. Blood.

[29] Meyer-Bahlburg A., Andrews S.F., Yu K.O., Porcelli S., Rawlings D.J. (2008). Characterization of a late transitional B cell population highly sensitive to BAFF-mediated homeostatic proliferation. J Exp Med.

[30] Prodeus A.P., Goerg S., Shen L.M., Pozdnyakova O.O., Chu L., Alicot E.M. (1998). A critical role for complement in maintenance of self-tolerance. Immunity.

[31] Kremlitzka M., Polgár A., Fülöp L., Kiss E., Poór G., Erdei A. (2013). Complement receptor type 1 (CR1, CD35) is a potent inhibitor of B-cell functions in rheumatoid arthritis patients. Int Immunol.

[32] Isnardi I., Ng Y.-S., Menard L., Meyers G., Saadoun D., Srdanovic I. (2010). Complement receptor 2/CD21- human naive B cells contain mostly autoreactive unresponsive clones. Blood.

[33] Rakhmanov M., Keller B., Gutenberger S., Foerster C., Hoenig M., Driessen G. (2009). Circulating CD21low B cells in common variable immunodeficiency resemble tissue homing, innate-like B cells. Proc Natl Acad Sci U S A.

[34] Saadoun D., Terrier B., Bannock J., Vazquez T., Massad C., Kang I. (2013). Expansion of autoreactive unresponsive CD21-/low B cells in Sjögren's syndrome-associated lymphoproliferation. Arthritis Rheum.

[35] Cancro M.P. (2009). Signalling crosstalk in B cells: managing worth and need. Nat Rev Immunol.

[36] Meffre E. (2011). The establishment of early B cell tolerance in humans: lessons from primary immunodeficiency diseases. Ann N Y Acad Sci.

[37] Westerberg L.S., Dahlberg C., Baptista M., Moran C.J., Detre C., Keszei M. (2012). Wiskott-Aldrich syndrome protein (WASP) and N-WASP are critical for peripheral B-cell development and function. Blood.

[38] Stepensky P., Krauss A., Goldstein G., Zaidman I., Elhasid R., Bielorai B. (2013). Impact of conditioning on outcome of hematopoietic stem cell transplantation for Wiskott-Aldrich syndrome. J Pediatr Hematol Oncol.

[39] Haddad E., Leroy S., Buckley R.H. (2013). B-cell reconstitution for SCID: should a conditioning regimen be used in SCID treatment?. J Allergy Clin Immunol.

[40] Tussiwand R., Rauch M., Flück L.A., Rolink A.G. (2012). BAFF-R expression correlates with positive selection of immature B cells. Eur J Immunol.

[41] Kreuzaler M., Rauch M., Salzer U., Birmelin J., Rizzi M., Grimbacher B. (2012). Soluble BAFF levels inversely correlate with peripheral B cell numbers and the expression of BAFF receptors. J Immunol.

[42] Groom J., Kalled S.L., Cutler A.H., Olson C., Woodcock S.A., Schneider P. (2002). Association of BAFF/BLyS overexpression and altered B cell differentiation with Sjögren’s syndrome. J Clin Invest.

[43] Becker-Merok A., Nikolaisen C., Nossent H.C. (2006). B-lymphocyte activating factor in systemic lupus erythematosus and rheumatoid arthritis in relation to autoantibody levels, disease measures and time. Lupus.

[44] Stohl W., Metyas S., Tan S.-M., Cheema G.S., Oamar B., Xu D. (2003). B lymphocyte stimulator overexpression in patients with systemic lupus erythematosus: longitudinal observations. Arthritis Rheum.

[45] Zhu X., Shi Y., Peng J., Guo C., Shan N., Qin P. (2009). The effects of BAFF and BAFF-R-Fc fusion protein in immune thrombocytopenia. Blood.

[46] Warnatz K., Wehr C., Dräger R., Schmidt S., Eibel H., Schlesier M. (2002). Expansion of CD19(hi)CD21(lo/neg) B cells in common variable immunodeficiency (CVID) patients with autoimmune cytopenia. Immunobiology.

[47] Brigida I., Sauer A.V., Ferrua F., Giannelli S., Scaramuzza S., Pistoia V. (2014). B-cell development and functions and therapeutic options in adenosine deaminase-deficient patients. J Allergy Clin Immunol.

[48] Jacobson C.A., Sun L., Kim H.T., McDonough S.M., Reynolds C.G., Schowalter M. (2014). Post-transplantation B cell activating factor and B cell recovery before onset of chronic graft-versus-host disease. Biol Blood Marrow Transplant.

[49] Tuveson D.A., Ahearn J.M., Matsumoto A.K., Fearon D.T. (1991). Molecular interactions of complement receptors on B lymphocytes: a CR1/CR2 complex distinct from the CR2/CD19 complex. J Exp Med.

